# Implicit motor imagery performance and cortical activity throughout the menstrual cycle

**DOI:** 10.1038/s41598-022-25766-2

**Published:** 2022-12-10

**Authors:** Rafaela Faustino Lacerda de Souza, Luana Adalice Borges de Araujo Lima, Thatiane Maria Almeida Silveira Mendes, Daniel Soares Brandão, Diego Andrés Laplagne, Maria Bernardete Cordeiro de Sousa

**Affiliations:** 1 Brain Institute, Federal University of Rio Grande do Norte, Rio Grande do Norte, 59078-970 Brazil; 2 Program in Psychobiology, Federal University of Rio Grande do Norte, Rio Grande do Norte, 59078-970 Brazil

**Keywords:** Motor control, Sensorimotor processing, Endocrinology, Human behaviour

## Abstract

Studies show that female motor and visuospatial skills are modulated by the menstrual cycle. Implicit motor imagery, meaning the involuntary imagination of movements during a task, involves kinesthetic, visual, and spatial aspects of the corresponding action and can be investigated by using the hand laterality judgment task (HLJT). In this study we aimed to investigate whether implicit motor imagery performance and cortical activity are altered throughout the menstrual cycle, as demonstrated by motor skills in females. Thus, 31 healthy women underwent HLJT during the menstrual, follicular and luteal phases of their menstrual cycles. Right-handed participants had to recognize the laterality (right or left) of hands presented in different views (palm or back) and orientations on a computer screen. Test performance and EEG event-related potentials were analyzed. Participants performed better in the test in the follicular and luteal phases when compared to the menstrual phase, and the accuracy of the test was positively correlated with estradiol levels in the follicular phase. The difference between medial and lateral hand orientations for rotation-related negativity was significant in the medial and left parieto-occipital regions only in the follicular phase. These findings suggest positive modulating action of estradiol in performing implicit motor imagery.

## Introduction

The fluctuation of sex steroids throughout the menstrual cycle is associated with variations in motor and visuospatial abilities seen in women in behavioral tests^1–6^. Better performance in motor activities that involve coordination and movement speed are observed in the follicular (where estrogen levels are high in the blood) and luteal (high levels of progesterone) phases when compared to the results observed in the menstrual phase (low estrogen and progesterone levels) of the menstrual cycle^1–4^. However, performance in activities involving visuospatial skills is better in the menstrual phase than those recorded in the follicular and luteal phases^3–6^.

Visuospatial skills can be investigated by the mental rotation test (MRT) of three-dimensional geometric figures. In this test, the individual must identify whether two similar objects in different orientations are identical or mirrored^7,8^. The time required to solve this task depends on the angular distance between the object’s orientations and requires the individual to mentally rotate the object in the same way as if the object physically existed^7^.

At a recent time, a study evaluated the influence of the estradiol on performance in MRTs, controlling for many confounding factors^6^. Lower estradiol levels were assessed by different control protocols, including checking menstrual cycle phase, using oral contraceptives, administrating testosterone hormone therapy to transmale women and comparing with male subjects. All of these groups showed that lower estradiol levels are associated with better performance on MRTs when compared with women in the menstrual cycle phases when estradiol is elevated. These observations provide strong evidence for the role of female hormones, in this case estradiol, in mental rotation.

When the object to be rotated in this type of test refers to parts of the body, such as the hands in the Hand Laterality Judgement Task (HLJT), it is common to use a strategy based on motor imagery to solve the test^9–12^. Although other strategies can be used to solve the HLJT, the motor strategy is the most efficient^10^.

During HLJT, the individual must identify whether the hands presented on a screen in different views (palm or back) and orientations are right or left^13^. In order to solve the task, the individual performs a mental transformation of their own hand in an attempt to find congruence with the hand presented in a process known as implicit motor imagery^12,14^. The time required to identify hands oriented towards the midline of the body (medial orientation) is longer than when compared to hands oriented away from the midline (lateral orientations). This is because lateral orientations biomechanically represent more difficult positions to be adopted, making the imagery take longer^9,15^. Also, the body posture itself influences the reaction time on the test^16–22^, supporting the hypothesis of using a motor strategy to solve the task.

Studies in which participants are asked to judge whether two hands presented simultaneously have similar or different laterality (a similar protocol to the MRT) have demonstrated better performance by women when compared to men^23,24^, suggesting that women adopt more motor strategies and men more visual and semantic strategies^24^. However, the effects observed in these studies were mild. In contrast, another study using the HLJT observed a difference between sexes with better performance for men during left hand recognition from the palm view in three orientations (0°, 90° and 180°, clockwise) and for women for left hand (0° and 90°) and right hand (0°) in the back view^25^. None of these studies were controlled for the menstrual cycle phase the women were in, which may have interfered with the disparity in reported results and this problem has been frequently repeated in studies looking at the effect of sex on MRTs and HLJT.

One important point to be taken into account is that motor imagery (implicit and explicit) is an important tool used for rehabilitation of neurological sequelae, as well as to improve motor performance skills^26–31^. Evidence indicates that the HLJT test is an accurate assessment tool for implicit motor imagery^32–36^. Despite this, there are no studies available on the possible actions of the female sex steroids in performing the HLJT test.

A recent study using a electroencephalographic (EEG) records showed that the frontal cortex is more activated during the performance of explicit motor imagery in the follicular phase when compared to other phases of the cycle of the menstrual^37^. However, there are no behavioral and neurophysiological correlates of the effect of the menstrual cycle on implicit motor imagery in literature.

EEG studies have revealed that HLJT triggers a positive component between 300 and 700 ms after presenting the hand in the event-related potential (ERP), known as the P300^19,38^. They also show a rotation related negativity (RRN), a negative component that is superimposed with the P300, that increases as rotation angles also increase^19,38^ and constitutes a neurophysiological correlate of implicit motor imagery^39^.

Therefore, the hypothesis of this study was that the motor strategy adopted for solving the HLJT task is favored in the follicular and luteal phases when compared to menstrual phase, as expected for performance on motor skills tests^1–4^. Thus, the aim of this study was to investigate the effect of the menstrual cycle on the behavioral performance of the HLJT (reaction time and accuracy) and its neurophysiological correlates (the EEG components evoked in the parieto-occipital region) by the stimulus characteristics (P100)^40–43^ and by implicit motor imagery (RRN)^19,38,39^.

## Methods

### Participants

A total of 206 women signed up to participate in the study. The inclusion criteria adopted for participants selection were: female sex, age between 18 and 30 years, regular menstrual cycle (between 22 and 35 days), and right-handedness. The exclusion criteria were: progesterone level < 10 nmol/l in the luteal phase as it is an indication of lack of ovulatory cycle^44,45^, pregnancy, history of neurological, psychological, immunological and/or endocrinological disorders, to have taken hormone-based medications in the last six months before the study began or psychotropic medication, primary hearing and/or visual impairment that cannot be improved with corrective lenses or hearing aids, and scores on the Mini-Exam of Mental State less than 24/25 points.

**Figure 1:**
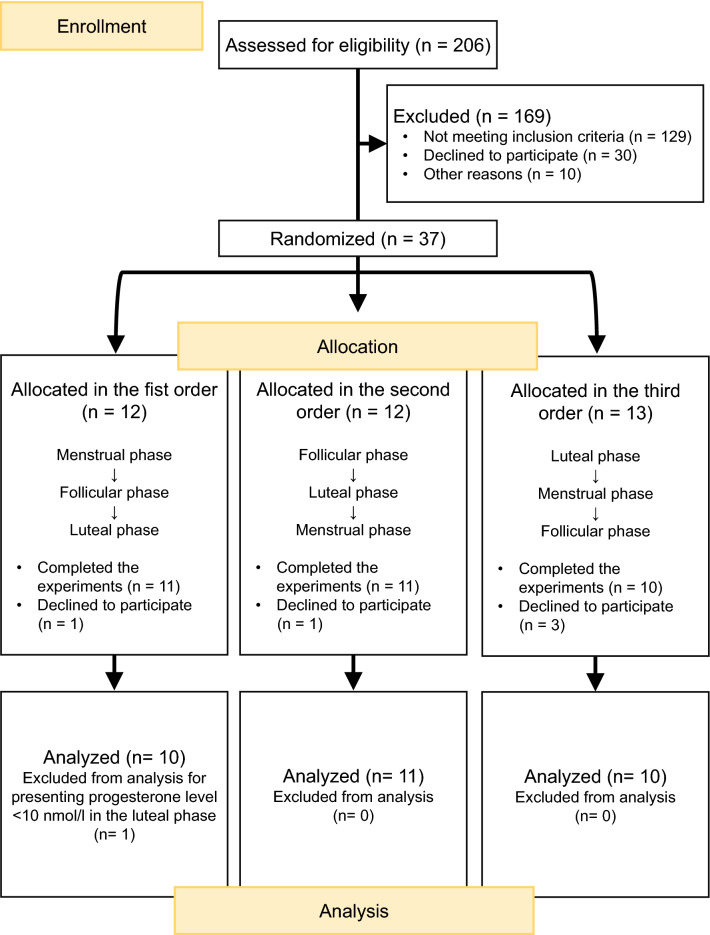
Flow diagram of the number of participants in enrollment, allocation and analysis phases of the study.

After eligibility assessment and initial screening, 169 women were excluded because they not met inclusion criteria or met the exclusion criteria (62.62% [129]), they refused to participate (14.56%^30^) or because it was not possible to contact them after registration (4.85%^10^). Among enrolled women who did not meet the inclusion criteria, 27.13% (35) for having an irregular menstrual cycle (> 35 days or < 22 days) and 0.77% (1) for being left-handed. Among those who met the exclusion criteria, 37.21% (48) reported having some type of psychological disorder (depression, anxiety disorder and panic syndrome), 36.43% (47) reported having endocrinological disorders (polycystic ovary syndrome, fibroids and endometriosis), 5.43% (7) reported having neurological disorders (history of seizures and chronic migraine), 0.77% (1) for reporting an autoimmune disease (systemic sclerosis), 3.88% (5) for using hormonal contraceptives and one participant (2.70%) was excluded after data collection for having low plasma progesterone levels. Of the 37 eligible participants who started the study, 13.51% (5) participants declined to continue due to time and day incompatibility (Fig. 1).

Finally, 31 women (mean age ± standard deviation: 25.0 ± 3.62, range 18–30 years) were included in the analysis of this study. They were followed-up for 6 months prior to starting the data collection to investigate the regularity and duration of their menstrual cycle (between 22 and 35 days)^46^ and the mean cycle length found was 28.6 ± 2.1 days. The duration of the menstrual cycle in this study was considered from the first day of menstrual bleeding in one cycle to the start of menstruation in the next cycle.

All participants were right-handed according to the Edinburgh Inventory^47,48^ and had no primary hearing or visual impairment that could not be corrected with the use of lenses. All participants signed an informed consent form to participate in the research project. The project was submitted and approved by the ethics committee of Federal University of Rio Grande do Norte (UFRN), Rio Grande do Norte, Brazil (CEP-UFRN, Process No. 2,519,458) and all experiments were performed in accordance with the ethical aspects related to research with human beings of Resolution No. 466/2012 of the National Health Council of Brazil. The project was also registered and approved by the Brazilian clinical trials registry (*Registro Brasileiro de Ensaios Clínicos*—REBEC) with the code: RBR-422dc96 and the Universal Trial Number (UTN): U1111-1263–1222.

### Experimental stimuli

**Figure 2:**
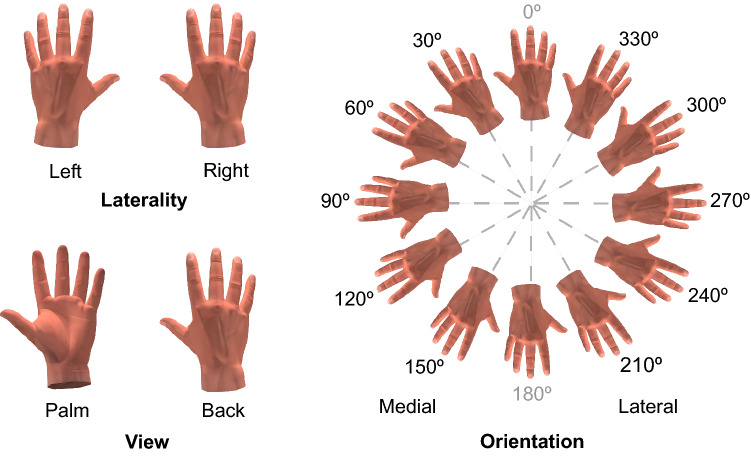
Experimental stimuli. Hand stimuli varied in terms of laterality, view and orientation.

The experimental stimuli were 3D images of both right and left hands with different views (back and palm). They were produced with Poser Pro^®^ 2014 software. Each of the four stimuli was oriented in 30° steps starting from 0° (fingertips up) to the 330° position resulting in 12 orientations. The right hands were oriented clockwise directions and the left hands were oriented in a counterclockwise direction. The hands oriented towards the midline of the body (with 30°, 60°, 90°, 120° and 150°) were classified as medial orientation and the hands in opposite direction (with 210°, 240°, 270°, 300° and 330°) as lateral orientation (Fig. 2). All stimuli were presented on a 21.5-inch LCD computer monitor positioned 50 cm away from the head.

### Study design

The participants were submitted to 5 protocols (Finger-Tapping Test, action observation, motor imagery, Go/No Go Test and HLJT) in a larger study. However, we only analyzed HLJT in this report to test the above mentioned hypothesis. Participants underwent 3 experimental sessions, preceded by a training session a week earlier. The participants learned and executed a block of the experimental protocols in the training session. Next, they were subjected to blood collection before engaging in the experimental protocols for electroencephalography (EEG) and audio recording. The experimental sessions took place in three different menstrual cycle phases (menstrual, follicular and luteal). The menstrual phase session was defined between days 2–5 from the beginning of menstruation. The follicular phase session was performed between days 9–12 of menstrual cycle for 28-day cycles. This interval varied proportionally according to the average duration of menstrual cycles. The luteal phase session took place from 7 to 9 days after the peak of the luteinizing hormone (LH). LH was monitored by the participants at home in daily urine samples using LH tests (Famivita/Innovita—Tangshan Biological Technology Co., Ltd., Hebei, China; sensitivity: 20 mUI/ml; product format: strip). Ten participants performed their first session when they were in the menstrual phase, eleven participants in the follicular phase and ten participants in the luteal phase to decrease order effects. The second and third sessions occurred in the two subsequent phases of the menstrual cycle (Fig. 1).

**Figure 3:**
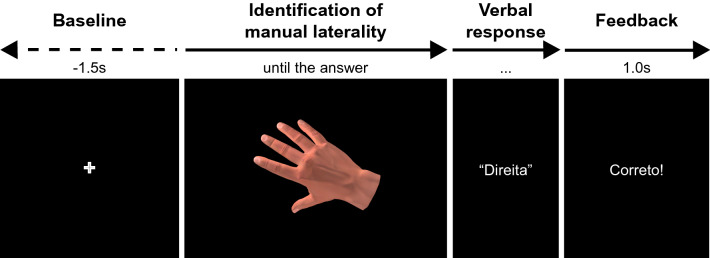
Sequence of events for the HLJT experimental protocol. First, a cross appeared on the screen. It was replaced by a hand stimulus. The hand remained on the screen until the verbal response of the participant. After the answer, feedback appeared on the screen indicating success or error.

The HLJT protocol was developed using Psychopy (v 1.73.04)^49,50^. It was applied in 3 blocks of 96 trials each, for a total of 288 trials. Participants were positioned seated in a comfortable cushioned chair and instructed to keep their hands relaxed with palms down on a wooden board. A cross appeared in the center of a black screen for 1.5 s in each trial, and the participants kept their eyes fixed on it. Then, the cross was replaced by the stimulus (one hand) and the participants had to identify whether the hand presented was right or left. They had to give a verbal response as quickly as possible. Then the experimenter pressed a button associated with the given answer. Finally, the stimulus was replaced by feedback indicating whether the answer was right or wrong (“*Correto*!” or “*Errado*!”, respectively). The feedback remained on the screen for 1 s, and then a new trial started (Fig. 3).

### ***Blood collection and hormonal assays***

For the hormonal assays, 10 mL of blood was collected in the morning between 8:00 and 9:00 am before placing the EEG electrodes in the 3 experimental sessions. After collection, the plasma was separated and kept in a freezer at − 20° Celsius. On the day of hormonal quantification, the samples were brought to room temperature and then the analytical procedures were performed. The chemiluminescent ELISA immunoassay technique was used and the progesterone and estradiol-17β measurements were performed using commercial kits from Immulite 2000^®^ (Progesterone Catalog Number: L2KPW2, analytical sensitivity 0.1 ng/mL [0.3 nmol/L]; Siemens Healthcare Diagnostics, Germany) and Access^®^ (estradiol-17β; kit Cat no B84493; Beckman-Coulter, Fullerton, CA, USA) kits, respectively. Intra-assay coefficient of variations was 8.36% ± 8.27% for progesterone and 3.20% ± 2.17% for estradiol, respectively.

### Data recording

Audio, EEG, and EMG data were synchronously and continuously recorded during the experimental sessions using a microphone, a 64-channel actiCap electrode cap, surface Ag/AgCl passive electrodes, BrainAmp DC amplifiers and the BrainVision Recorder version 1.20.0506 software program (Brain Products, GmbH). The sampling rate was set to 1000 Hz. EEG electrodes were positioned according to the 10–20 system and the electrical reference located in FCz. The electrode impedances were kept below 20 kΩ. To obtain the EMG, recording electrodes were positioned bilaterally over the extensor digitorum communis and ground electrode was placed on the right olecranon.

### Behavioral analysis

Reaction time and accuracy were considered in the behavioral analysis. The reaction time to HLJT was defined as the time interval between presenting the stimulus and the onset of verbal response. Audio data was used to define the onset of verbal response. To do so, it was segmented in epochs with intervals between 0 and 7 s relative to cross presentation and submitted to 8th order MA-whitening filter. These filtered epochs were submitted to the approximated generalized likelihood ratio (AGLR) decision rule based algorithm to detect abrupt changes in the variance of a signal and to define the onset of response^51^. Then, all trials with time markers of variance changes were subjected to visual and auditory inspection to select the time marker closest to the response onset. Trials with reaction times less than 500 ms and greater than 3500 ms were excluded (4.17%) from the behavioral and neurophysiological analysis^52,53^. Trials with incorrect answers were counted for accuracy analysis and removed from reaction time analysis (3.29%).

### Neurophysiological analysis

EEG and EMG data processing was performed using the EEGLAB toolbox version 13.6.5b^54^ in MATLAB (version 8.5, 2015a, The MathWorks Inc). EEG data was filtered between 1 and 40 Hz using the basic EEGLAB FIR filter, line noise was reduced using the CleanLine plugin and the bad channels with high impedances or displacement during recording were removed^55^. Independent component analysis (ICA) was performed and components containing eye, muscle or channel artifacts were removed automatically (20.82%) using ICLabel^56^. Then, the data was divided into segments from − 1 to 2 s relative to the appearance of the white cross on the screen. Epochs with voltage absolute values above 100 μV were subsequently removed (8.76%) and the channels removed before ICA were reconstructed by spherical interpolation (6.64%) and finally submitted to average reference. Epochs containing reaction times outside the range of 500–3500 ms (4.57%) or corresponding to incorrect answers (3.52%) were removed before ERP definition.

**Figure 4:**
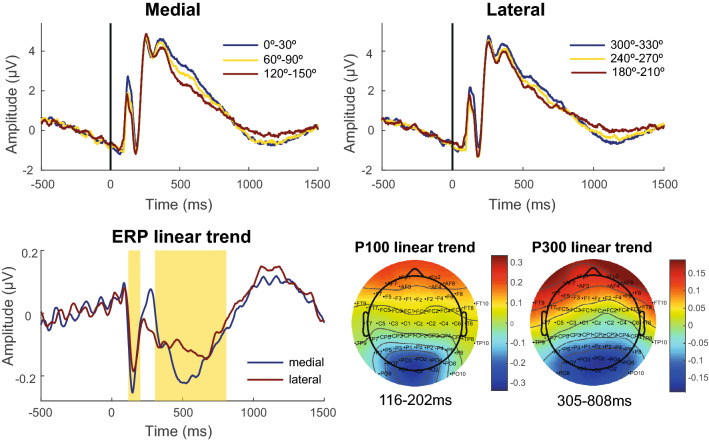
Defining window for ERP analysis. (**A**) Grand-average ERPs by double angles and orientations. (**B**) ERP linear trend. (**C**) Topography of the linear trend for each time interval defined for ERP analysis. The blue color represents the regions in which the ERP’s negativity increases due to the stimulus angles.

The ERP was obtained by the average of the segments grouped according to the stimuli characteristics (laterality, view and orientation). The time intervals for ERP analyses was defined from the linear trend between the 3 groups of angles for each orientation, following a method adapted from a previous study^57^. The grand-average ERPs of each group of orientation were multiplied by − 1, 0 and 1, respectively, and then divided by the sum square root of the squares of the constants (Fig. 4A). The linear contrasts of ERPs obtained in each orientation were added up to generate the linear trend ERPs. The time intervals were defined as 20% of the peaks found in the linear trend ERP (Fig. 4B). Two time intervals were defined, with one corresponding to the P100 component of the ERP (116–202 ms) and the other corresponding to the ERP RRN (305–808 ms). The topographic distribution of the linear trend ERP shows that the ERP negativity associated with increased angles is concentrated in the parieto-occipital region (Fig. 1C). Three cortical areas were investigated in this study: left parieto-occipital (P3, P5, P7, PO3, PO5, PO7, O1), medial parieto-occipital (P1, Pz, P2, POz, Oz) and right parieto-occipital (P4, P6, P8, PO4, PO6, PO8, O2).

EMG data was filtered between 10 and 499 Hz using the EEGLAB basic FIR filter, rectified and divided into epochs. From each epoch, two temporal windows were defined: the period of reference (from − 1 to 0 s) and the identification of laterality period (from 0 to the beginning of the verbal response, see Fig. 3). Root mean square (RMS) values of the EMG signal was calculated for each time window and then averaged per subject for both time windows to investigate the possible influence of EMG activity in the HLJT.

### Statistical analysis

Reaction time, accuracy, P100, and RRN amplitudes were statistically evaluated in principal analysis. The menstrual cycle phases (menstrual, follicular and luteal) and the stimulus features laterality (right and left), view (back and palm) and orientation (medial and lateral) were adopted as within-subject variables.

Statistical analyses were performed using the IBM SPSS software program (version 20.0; IBM Corp., NY, USA). The Friedman test was used to assess the estradiol and progesterone fluctuation throughout the 3 menstrual cycle phases. A generalized estimating equation (GEE)^58,59^ with an unstructured correlation matrix was adopted to test the effect of the within variables and interaction between them on the response time, accuracy, P100 and RRN amplitudes (in each parieto-occipital region), separately. To do so, the linear, gamma and tweedie distributions with the identity link function were tested for each dependent variable. The GEE model considering the gamma distribution obtained greater adherence based on the Quasi Likelihood under Independence Model Criterion (QIC). The GEE method produces regression estimates for repeated measures analysis or data clustered within subgroups with non-normal response variables^58,59^. Spearman’s correlation was used to verify the association between independent variables and hormone levels in the different menstrual cycle phases.

All p-values for analysis in various regions of the brain were presented after correcting for the number of cortical regions using the false discovery rate (FDR)^60^. Significant results involving factors with more than two levels or interactions were investigated by pairwise comparisons and p-value corrected by Bonferroni. The effect size of the comparisons was shown by the mean difference, Wald confidence interval of 95% of the mean difference and Cohen’s *d* estimate.

The RMS of EMG signal were compared between reference period and identification of laterality period in each menstrual cycle phase using Two-Sample Wilcoxon Signed Rank test. These analyzes were done in order to ensure that background EMG activity did not vary during HLJT.

All *p*-values < 0.05 were considered to indicate statistical significance.

## Results

### Estradiol and progesterone levels throughout the menstrual cycle

The hormonal profile of the menstrual cycle was characterized for all participants with variation in average values over the phases, varying as expected, with low plasma estradiol and progesterone levels in the menstrual phase, high estrogen levels in the follicular and luteal phases and high progesterone only in the luteal phase.

Significant variations in the plasma estradiol levels were found between the menstrual cycle phases (*χ*^2^ (2) = 43.16, *p* < 0.001, *W* = 0.70). Post hoc testing showed that the estradiol level significantly increased in the follicular (109.55 ± 81.34 pg/mL) and luteal (146.65 ± 45.83 pg/mL) phases compared to the menstrual phase (mean ± standard deviation = 33.11 ± 11.15 pg/mL, *p* < 0.001). No difference was found between the follicular and luteal phases for estradiol level (*p* = 0.067).

Plasma progesterone levels also significantly varied between the menstrual cycle phases (*χ*^2^ (2) = 47.92, *p* < 0.001, *W* = 0.77) and this hormone significantly increased in the luteal phase (10.03 ng/mL ± 4.21) compared to the menstrual (0.49 ± 0.27 ng/mL, *p* < 0.001) and follicular (0.47 ± 0.33 ng/mL, *p* < 0.001) phases. Progesterone levels in the menstrual and follicular phases were statistically similar (*p* = 0.92).

### Behavioral measures

#### Reaction time

**Figure 5:**
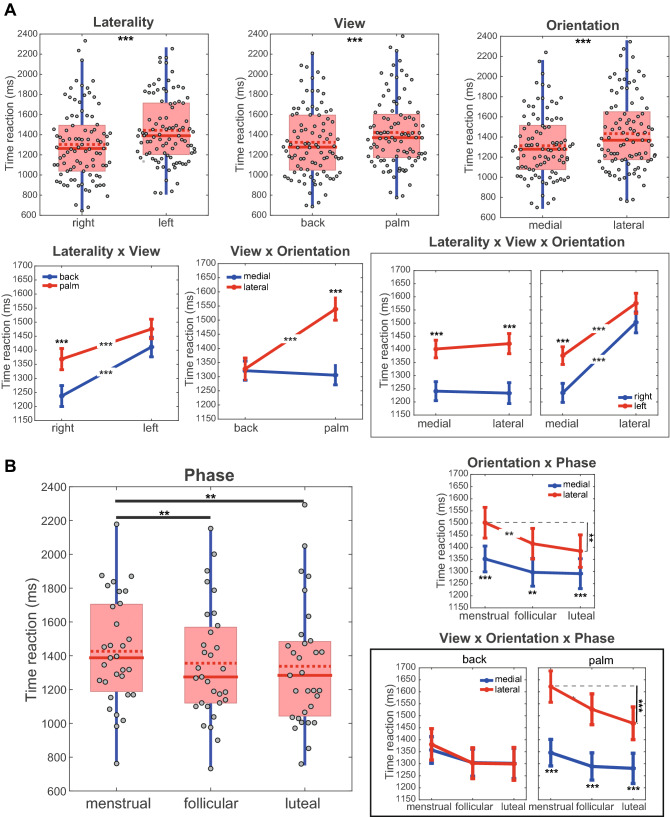
Interactions and main effects for reaction time. (**A**) Effect of hand features on the reaction time. (**B**) Effect of menstrual cycle and interaction between menstrual cycle and hand features on the reaction time. The main effects are presented in a boxplot. The filled red line in the boxplot represents the median and the dotted red line represents the mean. The interactions are presented in the form of mean and standard error. *P*-value: < 0.001 (***), < 0.01 (**), < 0.05 (*); Bonferroni correction for post-hoc tests.

The results related to the effects of the menstrual cycle phases *versus* stimuli features on the reaction time are shown in Fig. 5. The analysis revealed main laterality (χ^2^(1) = 76.77, *p* < 0.001), view (χ^2^ (2) = 32.41, *p* < 0.001), orientation (χ^2^(1) = 54.04, *p* < 0.001; Fig. 5A), and phase (χ^2^ (2) = 15.05, *p* < 0.001; Fig. 5B) effects. The right hand in the back view and medial orientation was recognized more quickly than the left hand (mean difference [95% Wald confidence interval for difference lower, upper] = − 140.76 ms [95% CI − 172, − 109.27], *d* = 0.43), in the palm view (− 97.80 ms [95% CI − 131.47, − 64.13], *d* = 0.30) and lateral orientation (− 120.09 ms [95% CI 152.11, − 88.07], *d* = 0.37), respectively. In the main effect analysis of the phase, the menstrual phase of the menstrual cycle showed a longer reaction time to HLJT and therefore worse performance than the follicular (70.81 ms [95% CI 21.53, 120.08], *p* = 0.002, *d* = 0.22) and luteal (88.85 ms [95% CI 21.58, 156.12], *p* = 0.005, *d* = 0.26) phases.

The analysis only for the characteristics of the stimuli found significant interactions for laterality x visualization x view (*χ*^2^ (1) = 18.29, *p* < 0.001), view x orientation (*χ*^2^ (1) = 57.92, *p* < 0.001), laterality x view x orientation (*χ*^2^ (1) = 6.99, *p* = 0.008) and is illustrated in Fig. 5A. The right hand in the back view was recognized more quickly than the right hand in the palm view (− 131.42 ms [95% CI − 186.53, − 76.32], *p* < 0.001, *d* = 0.38) and left hand in both back (− 174.38 ms [95% CI − 224.50, 124.25], *p* < 0.001, *d* = 0.52) and palm (− 238.56 ms [95% CI − 302.33, − 174.79], *p* < 0.001, *d* = 0.72) views. There was no difference in reaction time between medial and lateral orientations in the back view (6.42 ms [95% CI − 42.34, 55.18], *p* = 1), *d* = 0.02). This difference only appears in the palm view (− 233.76 ms [95% CI − 300.42, − 167.10], *p* < 0.001, *d* = 0.70). Left hand identification in palmar view and lateral orientation and in back view with any orientation had a longer reaction time when compared to the right hand (*all ps* < 0.05) (Fig. 5A).

The interactions between orientation x phase (*χ*^2^ (2) = 7.03, *p* = 0.033) and view x orientation x phase (*χ*^2^ (2) = 6.99, *p* = 0.03) also had significant effects (Fig. 5B). It was possible to observe differences between the lateral and medial orientations for the menstrual (149.36 ms [95% CI 72.82, 225.89], *p* < 0.001, *d* = 0.46), follicular (118.02 ms [95% CI 73.61, 162.44], *p* = 0.005, *d* = 0.35) and luteal phases (92.89 ms [95% CI 43.67, 142.11], *p* < 0.001, *d* = 0.26). The reaction time for lateral orientation was significantly longer in the menstrual phase than in the follicular (86.47 ms [95% CI 15.53, 157.42], *p* = 0.005, *d* = 0.24) and luteal phases (117.08 ms [95% CI 32.94, 201.22], *p* = 0.001, *d* = 0.32). No difference was found between the three menstrual cycle phases for medial orientation (all *ps* > 0.18). Differences between the lateral and medial orientations were only found for the palm view in the menstrual (275.32 ms [95% CI 146.31, 404.33], *p* < *0.001*, *d* = 0.80), follicular (238.18 ms [95% CI 164.51, 311.84], *p* < 0.001, *d* = 0.70) and luteal phases (187.79 ms [95% CI 103.40, 272.18], *p* < 0.001, *d* = 0.51). No difference between the orientations and the menstrual cycle phases was observed for the back view (all *ps* > 0.30). The reaction time for hand recognition for palm view and lateral orientation in the menstrual phase was significantly longer than in the follicular (94.43 ms [95% CI 2.05, 186.81], *p* = 0.038, *d* = 0.22) and luteal (152.79 ms [95% CI 34.56, 271.01], *p* < 0.001, *d* = 0.22) phases (Fig. 5B).

No significant hormonal associations were found for any phase of the menstrual cycle or for the grouping of all phases. Correlation analysis between reaction time and hormone levels was not significant (all *ps* > 0.05).

#### Accuracy

**Figure 6:**
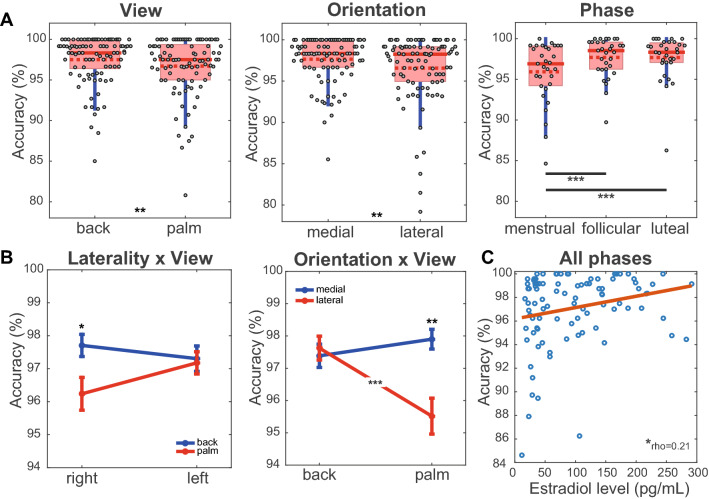
Effects of hand features and menstrual cycle on the accuracy. (**A**) Main effect of hand features on the accuracy. (**B**) Effect of interaction between hand features on the accuracy. (**C**) Correlation between accuracy and estradiol level. The main effects are presented in a boxplot. The filled red line in the boxplot represents the median and the dotted red line represents the mean. The interactions are presented as mean and standard error. *P*-value: < 0.001 (***), < 0.01 (**), < 0.05 (*); Bonferroni correction for post-hoc tests.

The results for menstrual cycle phases and hand precision features on accuracy are shown in Fig. 6. The main effects were significant for view (*χ*^2^ (1) = 8.13, *p* = 0.004), orientation (*χ*^2^ (1) = 8.02, *p* = 0.005) and phase (*χ*^2^ (2) = 22.06, *p* < 0.001) (Fig. 6A). The accuracy to identify the laterality of the hands in the back view was higher than the hands in the palm view (0.80% [95% CI 0.25, 1.34], *p* = 0.004, *d* = 0.28). Medially oriented hands were more accurately identified than laterally oriented hands (− 1.07% [95% CI − 1.81, − 0.33], *d* = 0.37). The accuracies were higher in the follicular phase (− 1.78% [95% CI − 2.67, − 0.73], *p* < 0.001, d = 0.57) as well as the luteal phase (− 1.76% [95% CI − 2.67, − 0.87], *p* < 0.001, d = 0.55) than in the menstrual phase.

The interactions between laterality x view and view x orientation were statistically significant (Fig. 6B). Identification of the right hands in the palm view was less accurate than that of the right hands in the back view (− 1.47% [95% CI − 2.72, − 0.21], *p* = 0.012, *d* = 0.42). The accuracy for identifying the left hands was the same for the back and palm views (− 0.53% [95% CI − 1.47, 0.41], *p* = 0.82, *d* = 0.05). Hands in palm view and lateral orientation showed lower accuracy when compared to medial palm (− 2.38% [95% CI − 4.22, − 0.55], *p* = 0.004, *d* = 0.65), dorsolateral (− 2.11% [95% CI − 3.51, − 0.71], *p* < 0.001, *d* = 0.56) and dorsomedial (− 1.87% [95% CI − 3.20, − 0.54], *p* = 0.001, *d* = 0.50). No significant differences were found between medial and lateral orientations for back view accuracy (0.24% [95% CI − 0.66, 1.14], *p* = 1, *d* = 0.09).

Correlation analysis showed a significant association between the accuracy of the test and the estradiol levels considering the grouping of all menstrual cycle phases (rho = 0.21; *p* = 0.04; Fig. 6C). No significant correlation was observed between accuracy and estradiol level at any phase of the menstrual cycle (all *ps* < 0.05)”.

### Electrophysiological measures

#### Background EMG activity

No significant differences were found between reference period and identification of laterality period in different phases of the menstrual cycle for the average RMS of the right (menstrual [z = 0.59, *p* = 0.56, r = 0.02], follicular [z = 0.61, *p* = 0.54, r = 0.02], luteal [z = 0.68, *p* = 0.49, r = 0.03]) and left arm (menstrual [z = − 0.39, *p* = 0.69, r = 0.02], follicular [z = 0.06, *p* = 0.002, r = 0.02], luteal [z = − 1.47, *p* = 0.14, r = 0.06]).

#### P100

The statistical analysis of the P100 amplitude is shown in Table S5 of *supplementary materials*. No main effects were observed for this variable. The effect of the laterality x orientation interaction was significant for the left (χ^2^(1) = 12.09, *p* = 0.0013, *FDR corrected*) and right (χ^2^(1) = 5.31, *p* = 0.03, *FDR corrected*) parieto-occipital region. P100 amplitude was smaller during left hand recognition when compared to the right hand in lateral orientation (0.340 µV [95% CI 0.08, 0.60], *p* = 0.004, *d* = 0.19) in the left parieto-occipital region. It was also possible to differentiate the orientations for left hands with lower amplitude of P100 for lateral orientation (− 0.371 µV [95% CI − 0.57, − 0.17], *p* < 0.001, *d* = 0.20). No significant difference was observed in post hoc analysis for the right parieto-occipital region.

View x orientation interaction was significant for left (χ^2^(1) = 24.97, *p* < 0.001, *FDR corrected*), medial (χ^2^(1) = 36.87, *p* < 0.001, *FDR corrected*) and right (χ^2^(1) = 19.54, *p* < 0.001, *FDR corrected*) parieto-occipital regions. P100 amplitude was smaller for the lateral orientation when compared to the medial orientation for palm view in the left (− 0.401 µV [95% CI − 0.65, − 0.16], p < 0.001, d = 0.21), medial (− 0.477 µV [95% CI − 0.70, − 0.25], *p* < 0.001, d = 0.32) and right (− 0.394 µV [95% CI − 0.63, − 0.15], *p* < 0.001, d = 0.23) parieto-occipital regions. The opposite (i.e. higher P100 amplitude) was observed for the back view on the left (0.289 µV [95% CI 0.05, 0.53], *p* = 0.01, d = 0.15), medial (0.288 µV [95% CI 0.11, 0.47], *p* < 0.001, d = 0.20) and right (0.289 µV [95% CI 0.05, 0.53], *p* = 0.01, d = 0.16) parieto-occipital regions.

**Figure 7:**
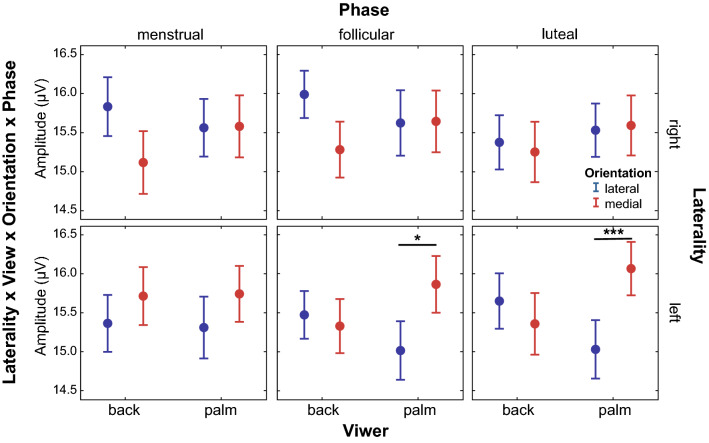
Effects of the interaction between laterality, view, orientation and phase on the P100 amplitude of the left parieto-occipital region. P100 amplitude differs between lateral and medial orientations for the left hands in the palm view during the follicular and luteal phases of the menstrual cycle. *P*-value: < 0.001 (***), < 0.01 (**), < 0.05 (*); Bonferroni correction for post-hoc tests.

The interaction between laterality, view, orientation and phase was significant for P100 amplitude in the left (χ^2^(2) = 14.83, *p* = 0.003, *FDR corrected*) and medial (χ^2^(2) = 7.31, *p* = 0.04, *FDR corrected*) parieto-occipital regions (Fig. 7). P100 in the left and medial parieto-occipital regions significantly varied depending on the orientation of the hand and the menstrual cycle phase for left hands in the palm view. The follicular (− 0.847 µV [95% CI − 1.62, − 0.07], p = 0.01, d = 0.42) and luteal (− 1.033 µV [95% CI − 1.75, − 0.32], *p* < 0.0001, d = 0.53) phases showed a smaller P100 amplitude for the lateral orientation than for the medial orientation in the left parieto-occipital region. A similar result was observed in the luteal phase (− 0.785 µV [95% CI − 1.52, − 0.05], *p* = 0.02, d = 0.47) in the medial parieto-occipital region. It was also possible to observe a difference in P100 amplitude in the luteal phase between the palm and back views (smaller in the palm) for the left hand in the medial orientation over the medial parieto-occipital region.

The correlation analysis performed for the three parieto-occipital regions did not show significant associations between the P100 amplitude and the variable estradiol and progesterone levels, or the progesterone/estradiol ratio (*all ps* > 0.05).

#### Rotation-related negativity (RRN)

**Figure 8:**
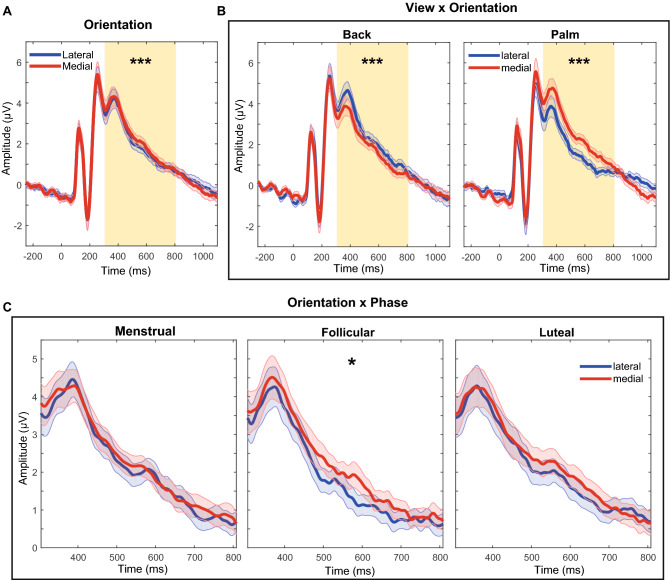
Effect of the menstrual cycle and hand features on ERP RRN in the left parieto-occipital region. (**A**) Main effect of orientation in ERP with higher RRN for lateral orientation than medial orientation. (**B**) View x orientation interaction effect on RRN. The RRN was higher for the lateral orientation then for the medial orientation for the palm view, the opposite was observed for the back view. The selected RRN window is highlighted by the yellow patch in the time interval between 305 and 808 ms after stimulus presentation (0 ms). (**C**) Orientation x phase interaction effect in the RRN window. The significant difference between the lateral and medial orientations was only observed for the follicular phase with a higher RRN for the lateral orientation than for medial orientation. RRN is represented in the time interval between 305 and 808 ms. *P*-value: < 0.001 (***), < 0.01 (**); < 0.05 (*); Bonferroni correction for post-hoc tests.

The effects of the menstrual cycle phases and hand features on RRN are shown for the left parieto-occipital region in Fig. 8.

The GEEs analysis of the RRN amplitudes revealed an orientation main effect in the left (χ^2^(1) = 14.88, *p* < 0.001, *FDR corrected*; Fig. 8A), medial (χ^2^(1) = 6.29, *p* = 0.012, *FDR corrected*) and right (χ^2^(1) = 7.93, *p* = 0.007, *FDR corrected*) parieto-occipital regions. RRN is higher during hand recognition in lateral orientations than in hands in medial orientations for the left (− 0.177 µV [95% CI − 0.27, − 0.09], *p* < 0.001, *d* = 0.16), medial (− 0.112 µV [95% CI − 0.20, − 0.02], *p* = 0.012, *d* = 0.11) and right (− 0.151 µV [95% CI − 0.26, − 0.05], *p* = 0.005, *d* = 0.11) parieto-occipital regions.

View x orientation interaction was significant for the left (χ^2^(1) = 55.23, *p* < 0.001; Fig. 8B), medial (χ^2^(1) = 43.07, *p* < 0.001) and right (χ^2^(1) = 37.44, *p* < 0.001) parieto-occipital regions. RRN is higher during palm hand recognition in lateral orientations than in medial orientations to the left (− 0.782 µV [95% CI − 1.06, − 0.51], *p* < 0.001, *d* = 0.69), medial (− 0.626 µV [95% CI − 0.90, − 0.35], *p* < 0.001, *d* = 0.60) and right (− 0.626 µV [95% CI − 0.90, − 0.35], *p* < 0.001, *d* = 0.48) regions. The opposite was observed during back hand recognition in which the RRN was higher for medial orientation than lateral orientation for the left (0.428 µV [95% CI 0.221, 0.64], *p* < 0.001, *d* = 0.36), medial (0.401 µV [95% CI 0.20, 0.60], *p* < 0.001, *d* = 0.36) and right (− 0.324 µV [95% CI 0.10, 0.55], *p* = 0.001, *d* = 0.23) regions.

The effect of orientation x phase interaction in RRN was significant for left (χ^2^(2) = 7.39, *p* = 0.037, *FDR corrected*; Fig. 8C) and right (χ^2^(2) = 7.99, *p* = 0.037, *FDR corrected*) parieto-occipital regions. The RRN was significantly higher for the lateral orientation than for medial orientation in the follicular phase for both the left (− 0.301 µV [95% CI − 0.46, − 0.14], *p* < 0.001, *d* = 0.24) and right regions (− 0.269 µV [95% CI − 0.43, − 0.10], *p* = 0.001, *d* = 0.19). No significant difference was observed between orientations in the menstrual (left: − 0.092 µV [95% CI − 0.32, 0.13], *p* = 1, *d* = 0.08 and right: − 0.139 µV [95% CI − 0.39, 0.11], *p* = 1, *d* = 0.09) and luteal (left: − 0.138 µV [95% CI − 0.31, 0.04], *p* = 0.35, *d* = 0.1 and right: − 0.044 µV [95% CI − 0.31, 0.22], *p* = 1, *d* = 0.03) phases.

The correlations between the RRN and the variable estradiol and progesterone levels and progesterone/estradiol ratio were not significant for the cycle phases or for any of the parieto-occipital regions (*all ps* > 0.05). The tables with all results can be found in the supplementary materials.

## Discussion

In general, it was possible to observe that our hypothesis that female sex steroids influence the motor strategy in the resolution of HLJT was confirmed, since the effect of the menstrual cycle on all outcomes studied was verified. A better test performance (shorter reaction time and greater accuracy) was observed in the follicular and luteal phases when compared to the menstrual phase. The electrophysiological analyzes show that in the initial phase of the visual analysis (P100) some characteristics are already differentiated in the follicular and luteal phases, suggesting the effect of estradiol alone and when associated to progesterone increase, respectively. These analyzes also showed that the strategy used in implicit motor imagery is remarkable in the follicular phase, since the RNN is significantly higher for lateral orientation when compared to medial (main marker of motor strategy), suggesting the effect of estradiol in this process.

The behavioral measurements, reaction time and accuracy, and the electrophysiological measurement, RRN of the ERP/EEG, are sensitive to the difficulty of adopting internal representations (implicit engine imagery) to solve the HLJT. On the other hand, the modulation of the component P100 of the ERP/EEG participates in the identification of the stimulus characteristics and may be involved in a visual analysis stage of the test.

The observed profiles regarding the analysis of the participants’ sex steroid levels validate what is expected for women with normal ovulation^61–65^. The highest estradiol concentrations were found in the follicular and luteal phases, and high progesterone levels were detected in the luteal phase, with these levels being significantly higher than in the menstrual phase.

The behavioral results of this study regardind reaction time and accuracy to identify laterality, orientation and view of the stimuli (except laterality for accuracy) reveal that the HLJT evokes in the participants the need to mentally simulate the movement. They also reveal that the reduced experience with the posture or the biomechanical restriction to adopt it can influence the performance of the test, as observed in the literature^9,15,66^. Right-handed participants recognized right hands more quickly than the left, probably because laterality generates a differentiation in the motor experience influencing the construction of internal representations^66^. In addition, medially oriented hands, with less biomechanical restriction^9,15^, were identified more quickly and accurately than laterally oriented hands.

Back view hands were identified faster and more accurately than palm view hands. One of the explanations suggested by the literature is that the back view is favored by visual familiarity and, therefore, a visual and non-motor strategy is adopted to identify these hands^25,39,53^. We suggest a new interpretation for this evidence based on the assumption that the motor imagery performed during HLJT is mainly kinesthetic, for which the movement is felt without the need to perform or visualize the movement^19^. This is because the forearm pronated with the hand positioned or not in front of the visual field for both medial orientations (e.g. when typing on a keyboard positioned in front of the body) and for lateral orientations (e.g. typing to reach an object located on the side of the body) is a more common proprioceptive experience than supinated forearm adopting the same orientations. The biggest biomechanical restrictions in the back position are for the fingertips angles downwards (i.e., 120°, 180° and 210°)^9^. Thus, viewing the palm is more suitable to investigate biomechanical restrictions and, consequently, the effect of motor imagery, when the medial and lateral orientation is considered^19,25,39,53^. This way of thinking also justifies the existence of an interaction between orientation and view for all outcomes, since there is only difference between orientations for palm view. It is important to consider that the visual analysis of the stimulus exists, but it occurs before the implicit motor imagery^38,40,41^.

These preliminary results are in agreement with what was observed in the literature and allow us to infer that the implicit motor imagery occurs regardless of the phase of the menstrual cycle. However, overall performance on the HLJT was significantly different between phases of the menstrual cycle, with shorter reaction times and greater accuracy observed during the follicular and luteal phases, when compared to menstrual phase. This difference is potentiated during the identification of hands in the lateral orientation and palm view (as seen, a view that favors biomechanical restriction considering medial and lateral orientations). This result suggests a positive effect of female steroids, especially estradiol which is high in these two phases, on performance in an implicit motor imagery test.

During the HLJT, individuals seek congruence between a hand presented on the computer screen and a mental representation of their own hand (right or left) that can be mentally moved^9–12^. Women perform better on similar tests in which congruence between objects (and not between hands) is sought in the menstrual phase than in the follicular and luteal phases^3–6^. Female performance in the menstrual phase in the mental rotation test of objects is similar to the performance of men, who perform better than women on this test when menstrual phases are mixed^6^. Thus, the findings of the present study show that spatial transformation is favored by female steroids when there is the possibility of adopting a motor strategy for solving the task to the detriment of the visuospatial strategy, commonly used in the mental rotation of objects.

Regarding the electrophysiological measures, EMG activity was monitored as a bias control measure. It did not differ between HLJT and reference periods, as expected since participants were instructed to keep their arms relaxed.

Apparently, the interaction between stimulus characteristics such as view and orientation in all parieto-occipital regions, as well as laterality and orientation in the lateralized regions (right and left parieto-occipitals) are evaluated in a visual processing step (by P100) that precedes the implicit motor imagery (by RRN) as already observed in other studies^38,40,68^. The clues acquired in the visual processing phase seem to indicate in which laterality and view of the hand (palm or back) should be mentally projected at the beginning of motor imagery. Interestingly, the visual processing stage in the follicular and luteal phases of the menstrual cycle is already capable of generating an assertive assumption about hand laterality in palmar view, which better differentiates medial and lateral orientations^19,25,39,53^. This finding was observed by EEG records in medial and left parieto-occipital regions and is in agreement with the behavioral results that associate the best performance in the test with the follicular and luteal phases. A previous study indicates that women have better initial visual processing than men and that this effect is stronger in the luteal phase of the menstrual cycle^68^.

The significant orientation effect was also observed in follicular phase of menstrual cycle for the RRN-ERP results in left and right parieto-occipital regions. This is interesting because the RRN in parieto-occipital regions is considered a marker of cognitive processing associated with motor imagery of the hand, since it varies in function of the biomechanical restrictions of the movement, and RRN is higher for hands in biomechanically more difficult positions to be adopted (lateral orientations)^38,67^. In follicular phase, RRN was higher for lateral than medial orientation in both regions, the electrophysiological evidence of participation of estradiol in facilitating the motor strategy for solving the task.

A positive correlation was found between accuracy and estradiol levels when measured from the three menstrual cycle phases together, suggesting a direct or indirect positive effect of high estradiol levels on test performance. Although the results provide much more evidence that implicit motor imagery is favored in the follicular and luteal phases, no significant correlation was found between the measures of reaction time, P100 and RRN amplitudes, and estradiol and progesterone levels, which suggests that the relationship between female steroids and cognitive motor processing may be being processed in other neural circuitry, subcortical or other cortical regions, involved in motor planning.

As observed in other studies, HLJT performance can be used to measure the ability to perform implicit motor imagery, as long as the learning effect is controlled^34^. Thus, the inclusion of a training session and the balance of the phases of the menstrual cycle were adopted as measures to control this effect in the present study.

Behavioral (reaction time and accuracy) and electrophysiological (P100 and RRN) measures showed evidence that the motor strategy used to recognize hand laterality in palm view can be favored by the increase in female steroids during the follicular and luteal phases of the menstrual cycle, as previously seen in coordination performance and manual dexterity test studies^1–4^. This evidence contributes to understanding the cyclic effect of steroid hormones on the motor control of women in reproductive age. Future studies can be carried out in order to investigate the association between menstrual cycle phases and motor learning potential.

## Supplementary Information

Supplementary Information.

## Data Availability

The datasets generated and analysed during the current paper are a part of a larger study. They can be made available through a formal agreement with the corresponding author.

## References

[1] Bayer U, Hausmann M (2012). Menstrual cycle-related changes of functional cerebral asymmetries in fine motor coordination. Brain Cognit..

[2] Zoghi M, Vaseghi B, Bastani A, Jaberzadeh S, Galea MP (2015). The effects of sex hormonal fluctuations during menstrual cycle on cortical excitability and manual dexterity (a pilot study). PLoS ONE.

[3] Hampson E, Kimura D (1988). Reciprocal effects of hormonal fluctuations on human motor and perceptual-spatial skills. Behav. Neurosci..

[4] Hampson E (1990). Estrogen-related variations in human spatial and articulatory-motor skills. Psychoneuroendocrinology.

[5] Hausmann M, Slabbekoorn D, Van Goozen SHM, Cohen-Kettenis PT, Güntürkün O (2000). Sex hormones affect spatial abilities during the menstrual cycle. Behav. Neurosci..

[6] Peragine D (2020). Sex difference or hormonal difference in mental rotation? The influence of ovarian milieu. Psychoneuroendocrinology.

[7] Shepard R, Metzler J (1971). Mental rotation of three-dimensional objects. Science (80-)..

[8] Vandenberg SG, Kuse AR (1978). Mental rotations, a group test of three-dimensional spatial visualization. Percept. Mot. Skills.

[9] Parsons LM (1994). Temporal and kinematic properties of motor behavior reflected in mentally simulated action. J. Exp. Psychol. Percept. Perform..

[10] Berneiser J, Jahn G, Grothe M, Lotze M (2018). From visual to motor strategies: Training in mental rotation of hands. Neuroimage.

[11] Kosslyn SM, Digirolamo GJ, Thompson WL, Alpert NM (1998). Mental rotation of objects versus hands: neural mechanisms revealed by positron emission tomography. Psychophysiology.

[12] Tomasino B, Gremese M (2016). Effects of stimulus type and strategy on mental rotation network: An activation likelihood estimation meta-analysis. Front. Hum. Neurosci..

[13] Cooper LA, Shepard RN (1975). Mental transformation in the identification of left and right hands. J. Exp. Psychol. Hum. Percept. Perform..

[14] Lebon F, Lotze M, Stinear CM, Byblow WD (2012). Task-dependent interaction between parietal and contralateral primary motor cortex during explicit versus implicit motor imagery. PLoS ONE.

[15] Parsons LM (1987). Imagined spatial transformations of one’s hands and feet. Cognit. Psychol..

[16] Ionta S, Sforza A, Funato M, Blanke O (2013). Anatomically plausible illusory posture affects mental rotation of body parts. Cogn. Affect. Behav. Neurosci..

[17] Ionta S, Fourkas AD, Fiorio M, Aglioti SM (2007). The influence of hands posture on mental rotation of hands and feet. Exp. Brain Res..

[18] de Lange FP, Helmich RC, Toni I (2006). Posture influences motor imagery: An fMRI study. Neuroimage.

[19] Jongsma MLA (2013). Effects of hand orientation on motor imagery—event related potentials suggest kinesthetic motor imagery to solve the hand laterality judgment task. PLoS ONE.

[20] Funk M, Brugger P (2008). Mental rotation of congenitally absent hands. J. Int. Neuropsychol. Soc..

[21] Shenton JT, Schwoebel J, Coslett HB (2004). Mental motor imagery and the body schema: Evidence for proprioceptive dominance. Neurosci. Lett..

[22] Sekiyama K (1982). Kinesthetic aspects of mental representations in the identification of left and right hands. Percept. Psychophys..

[23] Campbell MJ, Toth AJ, Brady N (2018). Illuminating sex differences in mental rotation using pupillometry. Biol. Psychol..

[24] Seurinck R, Vingerhoets G, De Lange FP, Achten E (2004). Does egocentric mental rotation elicit sex differences?. Neuroimage.

[25] Conson M (2020). Sex differences in implicit motor imagery: Evidence from the hand laterality task. Acta Psychol. (Amst).

[26] Zimmermann-Schlatter A, Schuster C, Puhan MA, Siekierka E, Steurer J (2008). Efficacy of motor imagery in post-stroke rehabilitation: A systematic review. J. Neuroeng. Rehabil..

[27] Yoxon E, Welsh TN (2019). Rapid motor cortical plasticity can be induced by motor imagery training. Neuropsychologia.

[28] Suso-Martí L, La Touche R, Angulo-Díaz-Parreño S, Cuenca-Martínez F (2020). Effectiveness of motor imagery and action observation training on musculoskeletal pain intensity: A systematic review and meta-analysis. Eur. J. Pain (U. K.).

[29] Nicholson V, Watts N, Chani Y, Keogh JW (2019). Motor imagery training improves balance and mobility outcomes in older adults: a systematic review. J. Physiother..

[30] Caligiore D, Mustile M, Spalletta G, Baldassarre G (2017). Action observation and motor imagery for rehabilitation in Parkinson’s disease: A systematic review and an integrative hypothesis. Neurosci. Biobehav. Rev..

[31] Aoyama T, Kaneko F, Kohno Y (2020). Motor imagery combined with action observation training optimized for individual motor skills further improves motor skills close to a plateau. Hum. Mov. Sci..

[32] McAvinue LP, Robertson IH (2008). Measuring motor imagery ability: A review. Eur. J. Cognit. Psychol..

[33] Kraeutner SN, Eppler SN, Stratas A, Boe SG (2020). Generate, maintain, manipulate? Exploring the multidimensional nature of motor imagery. Psychol. Sport Exerc..

[34] Boonstra AM (2012). Using the hand laterality judgement task to assess motor imagery: A study of practice effects in repeated measurements. Int. J. Rehabil. Res..

[35] de Vries S (2013). Motor imagery ability in stroke patients: the relationship between implicit and explicit motor imagery measures. Front. Hum. Neurosci..

[36] Breckenridge JD, Ginn KA, Wallwork SB, McAuley JH (2019). Do people with chronic musculoskeletal pain have impaired motor imagery? A meta-analytical systematic review of the left/right judgment task. J. Pain.

[37] Souza RFL (2022). Effect of the menstrual cycle on electroencephalogram alpha and beta bands during motor imagery and action observation. Front. Hum. Neurosci..

[38] ter Horst AC, Jongsma MLA, Janssen LK, Van Lier R, Steenbergen B (2011). Different mental rotation strategies reflected in the rotation related negativity. Psychophysiology.

[39] Zapparoli L (2014). Like the back of the (right) hand? A new fMRI look on the hand laterality task. Exp. Brain Res..

[40] Gootjes L, Bruggeling EC, Magnée T, Van Strien JW (2008). Sex differences in the latency of the late event-related potential mental rotation effect. NeuroReport.

[41] Heil M (2002). The functional significance of ERP effects during mental rotation. Psychophysiology.

[42] Mangun GR (1995). Neural mechanisms of visual selective attention. Psychophysiology.

[43] Herrmann CS, Knight RT (2001). Mechanisms of human attention: Event-related potentials and oscillations. Neurosci. Biobehav. Rev..

[44] Prior JC, Naess M, Langhammer A, Forsmo S (2015). Ovulation prevalence in women with spontaneous normal-length menstrual cycles - A population-based cohort from HUNT3, Norway. PLoS ONE.

[45] Bazanova OM, Nikolenko ED, Barry RJ (2017). Reactivity of alpha rhythms to eyes opening (the Berger effect) during menstrual cycle phases. Int. J. Psychophysiol..

[46] Creinin MD, Keverline S, Meyn LA (2004). How regular is regular? An analysis of menstrual cycle regularity. Contraception.

[47] Oldfield RC (1971). The assessment and Analysis of Handedness the Edinburgh Inventory. Neuropsychologia.

[48] Brito GNO, Brito LSO, Paumgartten FJR, Lins MFC (1989). Lateral Preferences in Brazilian Adults: An Analysis with the Edinburgh Inventory. Cortex.

[49] Peirce JW (2007). PsychoPy-Psychophysics software in Python. J. Neurosci. Methods.

[50] Peirce JW (2009). Generating stimuli for neuroscience using PsychoPy. Front. Neuroinform..

[51] Staude G, Kafka V, Wolf W (2000). Determination of promotor silent periods from suface myoelectric signal. Biomed. Tech.

[52] Ionta S, Perruchoud D, Draganski B, Blanke O (2012). Body context and posture affect mental imagery of hands. PLoS ONE.

[53] Bläsing B, Brugger P, Weigelt M, Schack T (2013). Does thumb posture influence the mental rotation of hands?. Neurosci. Lett..

[54] Delorme A, Makeig S (2004). EEGLAB: An open source toolbox for analysis of single-trial EEG dynamics including independent component analysis. J. Neurosci..

[55] Gabard-Durnam LJ, Leal ASM, Wilkinson CL, Levin AR (2018). The harvard automated processing pipeline for electroencephalography (HAPPE): Standardized processing software for developmental and high-artifact data. Front. Neurosci..

[56] Pion-Tonachini L, Kreutz-Delgado K, Makeig S (2019). ICLabel: An automated electroencephalographic independent component classifier, dataset, and website. Neuroimage.

[57] Milivojevic B, Hamm JP, Corballis MC (2009). Hemispheric dominance for mental rotation: It is a matter of time. NeuroReport.

[58] Liang KY, Zeger SL (1986). Longitudinal data analysis using generalized linear models. Biometrika.

[59] Muth C (2015). Alternative models for small samples in psychological research: Applying linear mixed effects models and generalized estimating equations to repeated measures data. Educ. Psychol. Meas..

[60] Benjamini Y, Hochberg Y, Benjamini, Yoav HY (1995). Controlling the false discovery rate: A practical and powerful approach to multiple testing. J. R. Stat. Soc..

[61] Fritz MA, Speroff L (2011). Clinical gynecologic endocrinology end infertility.

[62] Poromaa IS, Gingnell M (2014). Menstrual cycle influence on cognitive function and emotion processing from a reproductive perspective. Front. Neurosci..

[63] Schuster J, Karlsson T, Karlström P, Poromaa IS, Dahl N (2010). Down-regulation of progesterone receptor membrane component 1 ( PGRMC1) in peripheral nucleated blood cells associated with premature ovarian failure (POF) and polycystic ovary syndrome (PCOS). Reprod. Biol. Endocrinol..

[64] Verdonk SJE (2019). Estradiol reference intervals in women during the menstrual cycle, postmenopausal women and men using an LC-MS/MS method. Clin. Chim. Acta.

[65] Zhuang JY, Wang JX, Lei Q, Zhang W, Fan M (2020). Neural basis of increased cognitive control of impulsivity during the mid-luteal phase relative to the late follicular phase of the menstrual cycle. Front. Hum. Neurosci..

[66] Jones HG, Edwards FABLM, Conson RSCM (2021). The effect of handedness on mental rotation of hands: A systematic review and meta—analysis. Psychol. Res..

[67] Yu L (2020). Electrophysiological evidences for the rotational uncertainty effect in the hand mental rotation: An ERP and ERS/ERD study. Neuroscience.

[68] Lusk BR, Carr AR, Ranson VA, Felmingham KL (2017). Women in the midluteal phase of the menstrual cycle have difficulty suppressing the processing of negative emotional stimuli: An event-related potential study. Cogn. Affect. Behav. Neurosci..

