# Supplementary material for: Implicit motor imagery performance and cortical activity throughout the menstrual cycle
Source: Sci Rep. 2022 Dec 10;12:21385. doi: 10.1038/s41598-022-25766-2 (PMC9741588; doi:10.1038/s41598-022-25766-2)
Supplement: Supplementary file 1 — Supplementary Information. [file 41598_2022_25766_MOESM1_ESM.docx]

**Implicit motor imagery performance and cortical activity throughout the menstrual cycle**

**Supplementary materials**

Rafaela Faustino Lacerda de Souza^1*^, Luana Adalice Borges de Araujo Lima^1^, Thatiane Maria Almeida Silveira Mendes^1^, Daniel Soares Brandão^1^, Diego Andrés Laplagne^1^, Maria Bernardete Cordeiro de Sousa^1,2*^

^1^Brain Institute, Federal University of Rio Grande do Norte, Rio Grande do Norte, Brazil; ^2^Postgraduate Program in Psychobiology**,** Federal University of Rio Grande do Norte, Rio Grande do Norte, 59078-970, Brazil.

* mbcsousa@neuro.ufrn.br; rafaelafls@neuro.ufrn.br

## Behavioral measures

### Reaction time

**Table 1.** Interactions and main effects for reaction time

| Interactions | ꭓ2 | df | p | post hoc |
| --- | --- | --- | --- | --- |
| Laterality | **76.772***** | **1** | **<0.001** | right < left |
| View | **32.41***** | **1** | **<0.001** | back < palm |
| Orientation | **54.037***** | **1** | **<0.001** | medial < lateral |
| Phase | **15.05**** | **2** | **0.001** | follicular and luteal < menstrual |
| Laterality:View | **18.291***** | **1** | **<0.001** | left and back > others |
| Laterality:Orientation | 1.189 | 1 | 0.275 |  |
| Laterality:Phase | 0.22 | 2 | 0.896 |  |
| View:Orientation | **57.918***** | **1** | **<0.001** | palm: medial < lateral |
| View:Phases | 3.302 | 2 | 0.192 |  |
| Orientation:Phase | **7.034*** | **2** | **0.030** | lateral: menstrual > follicular, luteal |
| Laterality:View:Orientation | **6.994**** | **1** | **0.008** | left, palm and lateral > others |
| Laterality:View:Phase | 3.76 | 2 | 0.153 |  |
| Laterality:Orientation:Phase | 2.929 | 2 | 0.231 |  |
| View:Orientation:Phase | 6.993* | 2 | 0.030 | palm(lateral): follicular and luteal < menstrual |
| Laterality:View:Orientation:Phase | 2.865 | 2 | 0.239 |  |

P-value: < 0.001 (***), < 0.01 (**), < 0.05 (*), > 0.05 (ns); Bonferroni correction for post-hoc tests.

**Table 2.** Correlation between reaction time and hormonal level

| Phases | Estradiol | | Progesterone | | Progesterone/Estradiol | |
| --- | --- | --- | --- | --- | --- | --- |
|  | rho | p | rho | p | Rho | p |
| Menstrual | 0.127 | 0.496 | 0.042 | 0.824 | -0.073 | 0.698 |
| Follicular | 0.108 | 0.561 | 0.162 | 0.385 | -0.055 | 0.768 |
| Luteal | -0.045 | 0.811 | -0.003 | 0.989 | -0.046 | 0.808 |
| All phases | -0.055 | 0.6 | -0.024 | 0.817 | -0.057 | 0.589 |

### Accuracy

**Table 3.** Interactions and main effects for accuracy

| Interactions | ꭓ^2^ | df | p | post hoc |
| --- | --- | --- | --- | --- |
| Laterality | 0.634 | 1 | 0.426 |  |
| View | **8.126**** | **1** | **0.004** | back>palm |
| Orientation | **8.022**** | **1** | **0.005** | medial>lateral |
| Phase | **22.061***** | **2** | **<0.001** | follicular and luteal>menstrual |
| Laterality:View | **6.074*** | **1** | **0.014** |  |
| Laterality:Orientation | 0.293 | 1 | 0.588 |  |
| Laterality:Phase | 1.23 | 2 | 0.541 |  |
| View:Orientation | **10.977**** | **1** | **0.001** | palm: medial>lateral |
| View:Phases | 1.368 | 2 | 0.505 |  |
| Orientation:Phase | 2.387 | 2 | 0.303 |  |
| Laterality:View:Orientation | 3.143 | 1 | 0.076 |  |
| Laterality:View:Phase | 2.208 | 2 | 0.331 |  |
| Laterality:Orientation:Phase | 2.806 | 2 | 0.246 |  |
| View:Orientation:Phase | 3.205 | 2 | 0.201 |  |
| Laterality:View:Orientation:Phase | 1.876 | 2 | 0.391 |  |

P-value: < 0.001 (***), < 0.01 (**), < 0.05 (*), > 0.05 (ns); Bonferroni correction for post-hoc tests.

**Table 4.** Correlation between accuracy and hormonal level

| Phases | Estradiol | | Progesterone | | Progesterone/Estradiol | |
| --- | --- | --- | --- | --- | --- | --- |
|  | rho | p | rho | p | rho | p |
| Menstrual | 0.106 | 0.572 | -0.077 | 0.679 | -0.204 | 0.271 |
| Follicular | 0.033 | 0.86 | 0.144 | 0.438 | 0.046 | 0.805 |
| Luteal | 0.019 | 0.921 | 0.111 | 0.553 | 0.166 | 0.372 |
| All phases | **0.209*** | **0.044** | 0.14 | 0.182 | 0.044 | 0.677 |

P-value: < 0.001 (***), < 0.01 (**), < 0.05 (*).

## Electrophysiological measures

### P100

Table 5. Interactions and main effects for P100 amplitude

| Interactions | ꭓ2 | df | p | p-FDR | post hoc |
| --- | --- | --- | --- | --- | --- |
| Left parieto-occipital | | | | | |
| Laterality | 0.437 | 1 | 0.508 | 0.508 |  |
| View | 1.121 | 1 | 0.29 | 0.435 |  |
| Orientation | 1.287 | 1 | 0.257 | 0.283 |  |
| Phase | 0.16 | 2 | 0.923 | 0.923 |  |
| Laterality:View | 0.653 | 1 | 0.419 | 0.419 |  |
| Laterality:Orientation | **12.089**** | **1** | **0.001** | **0.003** | lateral: left < right  left: lateral < medial |
| Laterality:Phase | 7.02 | 2 | 0.03 | 0.09 |  |
| View:Orientation | **24.973***** | **1** | **<0.001** | **<0.001** | back: medial < lateral  palm: lateral < medial |
| View:Phases | 0.574 | 2 | 0.751 | 0.91 |  |
| Orientation:Phase | 1.598 | 2 | 0.45 | 0.675 |  |
| Laterality:View:Orientation | 1.125 | 1 | 0.289 | 0.289 |  |
| Laterality:View:Phase | 0.608 | 2 | 0.738 | 0.824 |  |
| Laterality:Orientation:Phase | 1.322 | 2 | 0.516 | 0.608 |  |
| View:Orientation:Phase | 3.924 | 2 | 0.141 | 0.336 |  |
| Laterality:View:Orientation:Phase | **14.83**** | **2** | **0.001** | **0.003** | left and palm (follicular and luteal): lateral < medial |
| Medial parieto-occipital | | | | | |
| Laterality | 0.73 | 1 | 0.393 | 0.508 |  |
| View | 1.366 | 1 | 0.242 | 0.435 |  |
| Orientation | 4.424 | 1 | 0.035 | 0.105 |  |
| Phase | 3.333 | 2 | 0.189 | 0.2835 |  |
| Laterality:View | 3.791 | 1 | 0.052 | 0.156 |  |
| Laterality:Orientation | 0.369 | 1 | 0.543 | 0.543 |  |
| Laterality:Phase | 2.054 | 2 | 0.358 | 0.358 |  |
| View:Orientation | **36.866***** | **1** | **<0.001** | **<0.001** | back: medial < lateral  palm: lateral < medial |
| View:Phases | 2.395 | 2 | 0.302 | 0.906 |  |
| Orientation:Phase | 0.093 | 2 | 0.955 | 0.955 |  |
| Laterality:View:Orientation | 3.147 | 1 | 0.076 | 0.201 |  |
| Laterality:View:Phase | 0.386 | 2 | 0.824 | 0.824 |  |
| Laterality:Orientation:Phase | 0.996 | 2 | 0.608 | 0.608 |  |
| View:Orientation:Phase | 2.991 | 2 | 0.224 | 0.336 |  |
| Laterality:View:Orientation:Phase | **7.31*** | **2** | **0.026** | **0.039** | left and palm (luteal): lateral < medial |
| Right parieto-occipital | | | | | |
| Laterality | 0.94 | 1 | 0.332 | 0.508 |  |
| View | 0.595 | 1 | 0.441 | 0.441 |  |
| Orientation | 1.153 | 1 | 0.283 | 0.283 |  |
| Phase | 7.089 | 2 | 0.029 | 0.087 |  |
| Laterality:View | 1.911 | 1 | 0.167 | 0.2505 |  |
| Laterality:Orientation | **5.315*** | **1** | **0.021** | **0.0315** | ns |
| Laterality:Phase | 3.249 | 2 | 0.197 | 0.2955 |  |
| View:Orientation | **19.545***** | **1** | **<0.001** | **<0.001** | back: medial < lateral  palm: lateral < medial |
| View:Phases | 0.188 | 2 | 0.91 | 0.91 |  |
| Orientation:Phase | 2.36 | 2 | 0.307 | 0.675 |  |
| Laterality:View:Orientation | 2.25 | 1 | 0.134 | 0.201 |  |
| Laterality:View:Phase | 1.741 | 2 | 0.419 | 0.824 |  |
| Laterality:Orientation:Phase | 1.974 | 2 | 0.373 | 0.608 |  |
| View:Orientation:Phase | 0.263 | 2 | 0.877 | 0.877 |  |
| Laterality:View:Orientation:Phase | 3.678 | 2 | 0.159 | 0.159 |  |

P-value: < 0.001 (***), < 0.01 (**), < 0.05 (*), > 0.05 (ns).

**Table 6**. Correlation between P100 amplitude and hormonal level

| Phases | Estradiol | | Progesterone | | Progesterone/Estradiol | |
| --- | --- | --- | --- | --- | --- | --- |
|  | rho | p | rho | p | rho | p |
| Left parieto-occipital | | | | | | |
| Menstrual | -0.206 | 0.267 | 0.032 | 0.864 | 0.147 | 0.431 |
| Follicular | -0.054 | 0.771 | -0.001 | 0.995 | -0.06 | 0.75 |
| Luteal | -0.242 | 0.19 | -0.181 | 0.331 | 0.02 | 0.914 |
| All phases | -0.096 | 0.359 | -0.047 | 0.651 | -0.008 | 0.943 |
| Medial parieto-occipital | | | | | | |
| Menstrual | -0.242 | 0.191 | -0.016 | 0.932 | 0.092 | 0.623 |
| Follicular | -0.003 | 0.986 | -0.077 | 0.681 | -0.076 | 0.685 |
| Luteal | -0.293 | 0.11 | -0.118 | 0.529 | 0.079 | 0.674 |
| All phases | -0.118 | 0.262 | -0.067 | 0.525 | -0.007 | 0.949 |
| Right parieto-occipital | | | | | | |
| Menstrual | -0.187 | 0.314 | -0.131 | 0.481 | 0.007 | 0.971 |
| Follicular | 0.204 | 0.271 | -0.084 | 0.651 | -0.252 | 0.171 |
| Luteal | -0.135 | 0.469 | 0.054 | 0.774 | 0.163 | 0.38 |
| All phases | 0.004 | 0.969 | -0.029 | 0.784 | 0.003 | 0.976 |

### RRN

Table 7. Interactions and main effects for RRN

| Interactions | ꭓ2 | df | p | p-FDR | | post hoc |
| --- | --- | --- | --- | --- | --- | --- |
| Left parieto-occipital | | | | | | |
| Laterality | 2.713 | 1 | 0.100 | 0.214 |  | |
| View | 0.007 | 1 | 0.931 | 0.931 |  | |
| Orientation | **14.882***** | **1** | **<0.001** | **<0.001** | lateral > medial | |
| Phase | 1.946 | 2 | 0.378 | 0.438 |  | |
| Laterality:View | 0.439 | 1 | 0.508 | 0.762 |  | |
| Laterality:Orientation | 2.029 | 1 | 0.154 | 0.462 |  | |
| Laterality:Phase | 1.516 | 2 | 0.469 | 0.7035 |  | |
| View:Orientation | **55.226***** | **1** | **<0.001** | **<0.001** | back: medial > lateral  palm: lateral > medial | |
| View:Phases | 1.901 | 2 | 0.386 | 0.386 |  | |
| Orientation:Phase | **7.386*** | **2** | **0.025** | **0.0375** | follicular: lateral > medial | |
| Laterality:View:Orientation | 5.184 | 1 | 0.023 | 0.069 |  | |
| Laterality:View:Phase | 1.036 | 2 | 0.596 | 0.596 |  | |
| Laterality:Orientation:Phase | 3.905 | 2 | 0.142 | 0.213 |  | |
| View:Orientation:Phase | 0.005 | 2 | 0.998 | 0.998 |  | |
| Laterality:View:Orientation:Phase | 1.545 | 2 | 0.462 | 0.737 |  | |
| Medial parieto-occipital | | | | | | |
| Laterality | 2.009 | 1 | 0.156 | 0.214 |  | |
| View | 1.366 | 1 | 0.243 | 0.3645 |  | |
| Orientation | **6.286*** | **1** | **0.012** | **0.012** | lateral > medial | |
| Phase | 2.037 | 2 | 0.361 | 0.438 |  | |
| Laterality:View | 1.093 | 1 | 0.296 | 0.762 |  | |
| Laterality:Orientation | 0.105 | 1 | 0.746 | 0.746 |  | |
| Laterality:Phase | 0.326 | 2 | 0.849 | 0.849 |  | |
| View:Orientation | **43.071***** | **1** | **<0.001** | **<0.001** | back: medial > lateral  palm: lateral>medial | |
| View:Phases | 5.569 | 2 | 0.062 | 0.1575 |  | |
| Orientation:Phase | 5.134 | 2 | 0.077 | 0.077 |  | |
| Laterality:View:Orientation | 0.349 | 1 | 0.555 | 0.555 |  | |
| Laterality:View:Phase | 3.526 | 2 | 0.172 | 0.258 |  | |
| Laterality:Orientation:Phase | 2.072 | 2 | 0.355 | 0.355 |  | |
| View:Orientation:Phase | 1.263 | 2 | 0.532 | 0.798 |  | |
| Laterality:View:Orientation:Phase | 0.611 | 2 | 0.737 | 0.737 |  | |
| Right parieto-occipital | | | | | | |
| Laterality | 1.543 | 1 | 0.214 | 0.214 |  | |
| View | 1.622 | 1 | 0.203 | 0.3645 |  | |
| Orientation | **7.933**** | **1** | **0.005** | **0.0075** | lateral > medial | |
| Phase | 1.651 | 2 | 0.438 | 0.438 |  | |
| Laterality:View | 0.027 | 1 | 0.869 | 0.869 |  | |
| Laterality:Orientation | 0.327 | 1 | 0.567 | 0.746 |  | |
| Laterality:Phase | 3.410 | 2 | 0.182 | 0.546 |  | |
| View:Orientation | **37.444***** | **1** | **<0.001** | **<0.001** | back: medial > lateral  palm: lateral > medial | |
| View:Phases | 4.506 | 2 | 0.105 | 0.1575 |  | |
| Orientation:Phase | **7.994*** | **2** | **0.018** | **0.0375** | follicular: lateral > medial | |
| Laterality:View:Orientation | 0.388 | 1 | 0.533 | 0.555 |  | |
| Laterality:View:Phase | 3.609 | 2 | 0.165 | 0.258 |  | |
| Laterality:Orientation:Phase | 4.199 | 2 | 0.123 | 0.213 |  | |
| View:Orientation:Phase | 2.163 | 2 | 0.339 | 0.798 |  | |
| Laterality:View:Orientation:Phase | 1.306 | 2 | 0.520 | 0.737 |  | |

P-value: < 0.001 (***). < 0.01 (**). < 0.05 (*). > 0.05 (ns).

**Table 8.** Correlation between RRN and hormonal level

| Phases | Estradiol | | Progesterone | | Progesterone/Estradiol | |
| --- | --- | --- | --- | --- | --- | --- |
|  | rho | p | rho | p | rho | p |
| Left parieto-occipital | | | | | | |
| Menstrual | -0.071 | 0.703 | -0.219 | 0.237 | -0.109 | 0.56 |
| Follicular | -0.082 | 0.66 | 0.02 | 0.915 | 0.069 | 0.714 |
| Luteal | 0.006 | 0.976 | 0.135 | 0.469 | 0.203 | 0.273 |
| All phases | -0.018 | 0.867 | 0.039 | 0.713 | 0.113 | 0.283 |
| Medial parieto-occipital | | | | | | |
| Menstrual | -0.129 | 0.488 | -0.269 | 0.143 | -0.045 | 0.809 |
| Follicular | -0.046 | 0.808 | -0.069 | 0.713 | 0.014 | 0.94 |
| Luteal | 0.074 | 0.693 | 0.154 | 0.409 | 0.146 | 0.433 |
| All phases | 0.024 | 0.822 | 0.008 | 0.941 | 0.062 | 0.553 |
| Right parieto-occipital | | | | | | |
| Menstrual | -0.14 | 0.452 | -0.353 | 0.051 | -0.146 | 0.432 |
| Follicular | 0.081 | 0.666 | -0.145 | 0.436 | -0.174 | 0.35 |
| Luteal | 0.227 | 0.219 | 0.118 | 0.528 | 0.103 | 0.581 |
| All phases | 0.06 | 0.57 | -0.073 | 0.488 | -0.027 | 0.794 |
